# Supplementary material for: DROSHA-Dependent AIM2 Inflammasome Activation Contributes to Lung Inflammation during Idiopathic Pulmonary Fibrosis
Source: Cells. 2019 Aug 20;8(8):938. doi: 10.3390/cells8080938 (PMC6721825; doi:10.3390/cells8080938)
Supplement: Supplementary file 1 [file cells-08-00938-s001.pdf]

Supplemental information

DROSHA-dependent AIM2 inflammasome activation contributes to lung inflammation during idiopathic pulmonary fibrosis.

Soo Jung Cho[1,2], Kyoung Sook Hong[1,2,3], Ji Hun Jeong[4], Mihye Lee[4], Augustine M. K. Choi[1,2], Heather W. Stout-Delgado[1,2] and Jong-Seok Moon[4*]

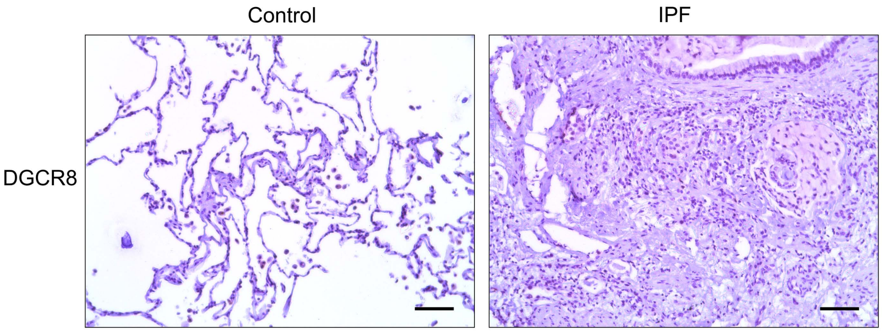

Supplemental Figure S1. DGCR8 expression is comparable in lung tissues from patients with IPF and healthy subject. Representative immunohistochemistry image of DGCR8 in lung tissues from patients with IPF (IPF) or healthy control (Control). Scale bar, 50 μM.

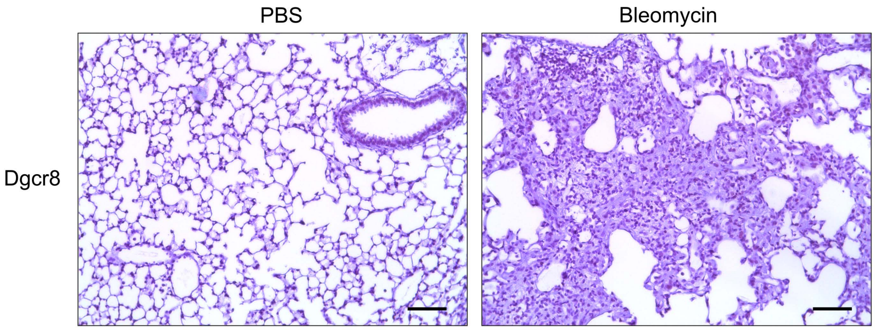

Supplemental Figure S2. DGCR8 expression is comparable in lung tissues during bleomycin-induced lung injury. Representative immunohistochemistry image of DGCR8 in lung tissues from WT mice were exposed to PBS ro belomycin via oropharyngeal aspiration. Scale bar, 50 μM.

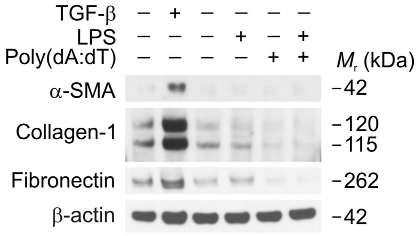

Supplemental Figure S3. LPS and poly(dA:dT) did not change the activation of fibroblasts. Representative immunoblot analysis for α-SMA, collagen-1 and fibronectin levels from mouse primary lung fibroblasts were treated with TGF-β (20 ng/ml, 24h), LPS or poly(dA:dT). For immunoblots, β-actin was used as loading control.
